# Green biosynthesis of silver and gold nanoparticles using Teak (*Tectona grandis*) leaf extract and its anticancer and antimicrobial activity

**DOI:** 10.1016/j.heliyon.2023.e21698

**Published:** 2023-11-04

**Authors:** Huda Mahdi Younis, Hanaa Ali Hussein, Fatin L. Khaphi, Zahra Kadhum Saeed

**Affiliations:** University of Basrah, College of Dentistry, Basrah, Iraq

**Keywords:** Teak extract, *Tectona grandis*, Silver nanoparticles, Gold nanoparticles, Green synthesis, Anticancer

## Abstract

The green synthesis of nanoparticles (NPs) utilizing a green path is eco-friendly and profitable compared to traditional physical and chemical techniques. This research conducted a green synthesis of gold NPs (AuNPs) and silver NPs (AgNPs) using an extract of Teak (*Tectona grandis*) and their anticancer and anti-microbial activities. Various techniques like transmission-electron microscopy (TEM), UV–Vis spectroscopy, thermal-gravimetric analyses (TGA), X-ray diffraction (XRD), and Fourier transform-infrared spectroscopy (FT-IR) were used to analyze synthesized AuNPs and AgNPs. The effects of different factors like the amount of extract used, solution pH, and contact time were measured to obtain the best possible conditions for synthesizing NPs. The AgNPs showed significant anticancer activity against HepG2 with an IC_50_ of 6.17 mg/ml compared to Teak extract (>50 mg/ml) and AuNPs (44.1 mg/ml), while AuNPs (6 % Teak extract and 2.9 × 10^−3^ M HAuCl_4_) showed significant antibacterial and antifungal activity against *Pseudomonas aeruginosa, Aspergillus niger*, *Bacillus subtilis*, and *Escherichia coli* with an inhibition zone of 11 mm, 12 mm, 12.5 mm, and 15.5 mm, respectively as compared to other treatments. These findings confirmed the medical applications of AuNPs and AgNPs and might open new possibilities in this field.

## 1 Introduction

Metal and metal oxide NPs (nanoparticles) have garnered significant interest in physical and chemical fields due to their distinct properties from bulk materials, their applications as delivery systems for drugs, biomolecules, antimicrobials, nucleic acids, and their roles in cancer diagnostics and treatment [[1], [2], [3], [4]].

**Fig. 1:**
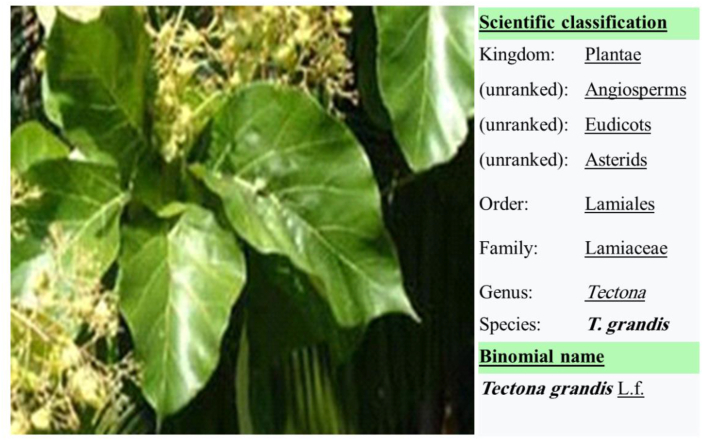
*Tectona grandis* leaf and its scientific classification.

Gold NPs (AuNPs) and silver NPs (AgNPs) can be synthesized through various methods, including physical (vapor deposition, lithography, thermal evaporation, and laser ablation) and chemical synthesis (evaporation solution method, sol-gel process, and vapor method). The most commonly used methods are photochemical reduction, chemical reduction, and electrochemical reduction. Chemical reduction involves reducing agents like citrate salt, sodium borohydride, elemental hydrogen, and ascorbate salt. While these agents are inexpensive, it is necessary to consider environmentally friendly alternatives like the Tollens, polysaccharide, irradiation, and biological (eco-friendly) methods. In the biological method, bio-organism extract can serve as reducing and capping agents for AuNPs and AgNP synthesis. The plant extract contains a bio-mixture of proteins, amino acids, vitamins, and polysaccharides that can reduce gold and silver to NPs [[5], [6], [7]]. Plants are a popular method for producing NPs due to their ease of preparation, effectiveness, and ability to produce large quantities of uncontaminated NPs with well-defined sizes and morphology (typically spherical). This makes them highly reactive and ideal for a wide variety of applications compared to other biological methods [8,9]. The disadvantage of using these methods is that the properties of the raw material restrict the range of conditions they can be utilized. This restriction can affect the formation of NPs. Therefore, it is crucial to establish clear specifications for metallic composition, temperature, reaction time, and pH [10]. Numerous studies have explored the biosynthesis of AuNPs and AgNPs using plant leaf extracts, but there has been little research on the green synthesis of AgNPs and AuNPs using *Tectona grandis* that exhibit potential anticancer and antibacterial activity. The Teak tree, also known as *Tectona grandis*, is part of the Lamiaceae family (formerly known as Verbenaceae). This family is well-known for its aromatic members such as basil, oregano, and rosemary, which are commonly used in Southeast Asian cuisine (Fig. 1).

AuNPs are an excellent material for study, as they are non-toxic, stable, and ease to prepare. Green synthesis of AuNPs has been previously reported in several studies. For example, AuNPs prepared using *Terminalia catappa* (leaf extract) were spherical with a size of 21.9 nm [11], AuNPs with a particle size of 15 ± 5 nm and spherical shape were prepared using *Crocus sativus* [12], AuNPs with hexagonal triangle, truncated triangle, and spherical shapes and a particle size of 20–42 nm and 62–88 nm synthesized using *Justicia gendarussa* (leaf extract) [13], AuNPs with triangular, hexagonal, and spherical shapes and a particle size of 10–55 nm prepared using *Cassia auriculata* flower extract [14], and AuNPs with a particle size of 5.82 nm and spherical shape synthesized using *Clerodendrum inerme* leaf extracts [8]. Research has preferred AgNPs due to their special chemical, biological, and physical features. The AgNP surface chemistry, shape, and size serve a key function in the biological activity of AgNPs [15]. The literature has previously reported green production of AgNPs using *Clerodendrum inerme* were a size of 5.54 nm and spherical [8], AgNPs with a particle size of 10–30 nm and oval or spherical shape synthesized using *Tectona grandis* seeds extract, NPs were [16], and AgNPs synthesized using *Momordica cymbalaria* (fruit extract), the NPs were with a size of 15.5 nm and spherical [17].

In previous studies, AuNPs and AgNPs were used as anticancer agents against different types of cancer cells. For example, AgNPs synthesized using *Cucumis prophetarum* (aqueous leaf extract) exhibited cytotoxic activity in MCF-7, MDA-MB-231, HepG2, and A549 cells with a half-maximal inhibitory concentration (IC_50_) value of 65.6, 81.1, 94.2, and 105.8 μg/ml, respectively [18]. Meanwhile, AgNPs synthesized using palm extract showed anti-cancer activity in PC3 cells with an IC_50_ of 8.1 μg/ml [9]. Similarly, Brazilian red propolis-mediated synthesis AuNPs displayed cytotoxicity in PC-3 and T24 cells with IC_50_ values of 53 and 43.1 mg/ml, respectively [19]. On the other hand, others reported that the AgNPs synthesized using *Amorphophallus paeoniifolius* exhibited the highest activity against *Pseudomonas aeruginosa* with an inhibition zone of 20 nm [20,21]. Francis et al. [22] reported that *Stereospermum chelonoides*-mediated synthesis of AuNPs promoted the highest activity against *Aspergillus flavus* with an inhibition zone of 21 mm.

Therefore, the present study evaluated the nontoxic, simple, and rapid method for the green and stable production of Ag and Au NPs using Teak extracts, and study characterization was carried out by transmission-electron microscopy (TEM), UV–Vis spectroscopy, thermal-gravimetric analyses (TGA), X-ray diffraction (XRD), and Fourier transform-infrared spectroscopy (FT-IR) analysis; *in-vitro* anti-cancer against liver cancer cell-lines HepG2 and antimicrobial activities against gram-positive (*Bacillus subtilis* and *Escherichia coli)*, gram-negative (*P. aeruginosa* and *Staphylococcus aureus*) bacteria, and fungi (*Aspergillus niger* and *Candida albicans*) were examined.

## 2 Materials and methods

### 2.1 Materials and chemicals

Teak leaves were gathered from the Orman Garden in Giza, Egypt. Sigma-Aldrich USA provided the gold (HAuCl_4_.H_2_O, 99.9 %), silver nitrate (AgNO_3_, 99.9 %), sulphuric acid (H_2_SO_4_), sodium hydroxide (NaOH), and hydrochloric acid (HCl) utilized for monitoring the pH.

### 2.2 The extraction steps

To prepare the extract, 4.0 g of fresh and healthy teak leaves were cleaned thoroughly with distilled water (D.W.) to eliminate any particles present on their surface. The leaves were then heated for 15 min, and the resulting extract was filtered using filter paper. The filtered extract was then made up to 100 ml with D.W. to achieve a 4 % w/v extract. The pH of the solution was adjusted using 0.1 N HCl and NaOH solutions to prepare the NPs at different pH levels.

### 2.3 Synthesis of NPs

A gold stock solution was created by dissolving HAuCl_4_. H_2_O (1 g) in 50 ml D.W. in dark conditions. AgNO_3_ (1 × 10^−2^ M) was created for the silver stock solution by dissolving 0.084 g in 50 D.W. For AuNPs, Teak extracts at a concentration of 4 % (w/v) were added at room temperature to the 0.05 ml HAuCl_4_.H_2_O solution, and the volume was completed to 10 ml with D.W. Therefore, the concentration of Au^3+^ was 2.9 × 10^−4^ M, and the reduction method of Au^3+^ to AuNPs was confirmed by a color change from yellow to green. For AgNPs, 0.5 ml of the Teak extracts was loaded to the 0.1 ml AgNO_3_ solution and the volume was completed to 10 ml with D.W. The concentration of Ag^+^ was 1 × 10^−4^ M, and the reduction of Ag^+^ to AgNPs was confirmed by a color change from yellow to dark brown within 24 h.

### 2.4 Characterizations studies

UV–Vis spectra were obtained using a spectrophotometer 2600 (Shimadzu) at room temperature. XRD pattern was attained utilizing an XRD 6000-diffractometer with Cu Ka (Shimadzu, *λ* = 1.54056 Å) to confirm the NPs’ biosynthesis. UV–Vis and XRD were described before by Hussein et al. [23]. The morphology and size of the NPs were determined using TEM (1200JEM-JEOL). An FTIR spectrometer (Nicolet 6700) recorded the FTIR spectra at room temperature. TGA analysis was conducted using a DT-50 thermal analyzer with a heating rate of 10 °C/min (Shimadzu). TEM, DLS, FTIR, and TGA were as described before by Khalil et al. [24].

### 2.5 Cytotoxicity activity

*In-vitro* anti-cancer activity of the NPs was performed against HePG2 (hepatocellular carcinoma) cell lines using the MTT (3-(4,5-Dimethylthiazol-2-yl)-2,5-diphenyltetrazolium bromide) method. The MTT assay was performed following the protocol by Hussein et al. [25]. Doxorubicin-hydrochloride (DOX-Hcl) is among the highly effective anticancer drugs and was used in this study as a positive control.

### 2.6 Antimicrobial activity

Two stock plant extracts (1.2 % and 6 % w/v) were prepared by boiling 4 g and 20 g of the extracts separately for 15 min. After filtering, the volume of each extract was completed to 100 ml with D.W. A 2 ml of stock solution (1.2 % and 6 % w/v) was utilized and diluted to 10 ml with D.W. Different concentrations of extracts, Au^3+^, and Ag^+^ were prepared as follow: 1.2 % extract only; 6 % extract only; AuNPs (1.2 % extract and 1.45 × 10^−3^ M Au^3+^); AuNPs (6 % extract and 2.9 × 10^−3^ M Au^3+^); AgNPs (1.2 % extract and 5 × 10^−3^ M Ag^+^); and AgNPs (6 % extract and 1 × 10^−4^ M Ag^+^). The antimicrobial activity was tested using the well-diffusion process, as previously reported by Khalil et al. [24] and Hussein et al. [26].

## 3 Results and discussion

### 3.1 Characterization of NPs

The first indication of the NP's formation is altering the reaction mixture's color. The colorless solution of HAuCl_4_ was rapidly converted from yellow into mauve, purple, and green following the AuNPs shape and size, which indicated the bio-reduction of Au^3+^, as reported by Kuthi et al. [27]. The mixture changed from colorless to dark brownish-black due to the rapid formation of AgNPs, which can be attributed to the excitation of surface plasmon vibration in the AgNPs. A similar visual observation was found in AgNPs synthesized from *Prosopis juliflora* extract [28].

### 3.2 UV–Vis spectrophotometer analysis

**Fig. 2:**
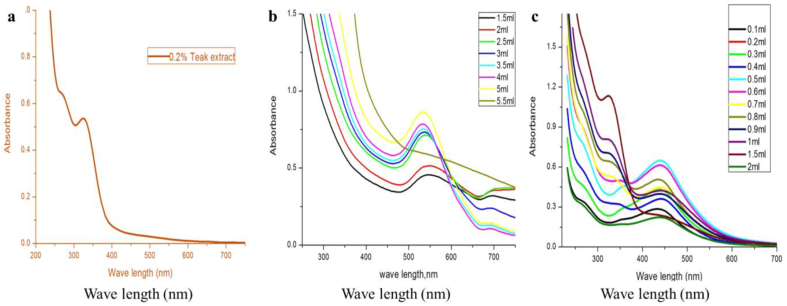
UV–Vis spectra of (a) 0.2 % of the Teak extracts; (b) AuNPs; (c) AgNPs at different concentrations of Teak leaf extract.

Fig. 2a shows the absorption spectrum of 0.2 % *Tectona grandis* extract. The chemical constituents in the aqueous extract exhibit a strong band at around 340 nm and a shoulder at around 280 nm. These bands are due to n- π* and π- π* and suggest the presence of organic compounds in the Teak extract. For the efficient synthesis of AgNPs and AuNPs, various parameters were taken into account, including the amount of extract, contact time, pH, and temperature, were optimized. The amount of Teak extract employed is an important parameter for regulatory secondary plant metabolites and determining the size of the NP cluster. Fig. 2 b shows the AuNP's UV–Vis spectra using different extract concentrations. Broad-band was observed at low concentrations of less than 0.9 ml (data not shown) without a distinguished maximum peak. The absorption spectra exhibited a gradual growth in absorbance with increasing extract concentration from 1 ml to up to 5 ml, along with a shift in the *λ*_max_ from 535 to 550 nm. The absorption maximum at 535–550 nm may be attributed to the surface plasmon resonance band (SPR) of the AuNPs. A peak was observed at 700 nm with intensity decreased and increasing extract amount to 5 ml. This band suggests the presence of aggregation of the NPs. At more than 5 ml, the absorption was decreased, and again, there was no clear peak, and the solution color turned green, indicating saturation had been reached in the Au^3+^ bioreduction using 5 ml. Thus, the peak at 535 nm confirmed the formation of AuNPs. Akilandaeaswari and Muthu [29] and Khan et al. [8] reported a similar absorption spectrum at peak 546 nm and 534 nm, respectively, for AuNPs. A previous study reported that the supreme absorption is red-shifted as the NP size increases. Thus, the conclusion can be reached that an NP's size decreases as the concentration of the extract increases from 1.5 to 5 ml as the absorption spectra are blue-shifted [27]. In contrast, AuNPs synthesized with Gmelina extract showed a clear band at a concentration of 0.9 ml compared to 0.2 ml of Gemlina extract. This result indicates that the reducing compounds in *Gmelina arborea* extract are more active than those in Tecona extract [24]. Fig. 2c shows the UV–Vis spectra of AgNP at absorbance 440–456 nm. A peak detected at 300–500 nm confirmed the formation of AgNPs [30]. As the leaf extract concentration increases (from 0.1 to 2 ml), a blue shift was detected from 456 to 440 nm in the spectra. This blue shift shows a decrease in the AgNP's size.

**Fig. 3:**
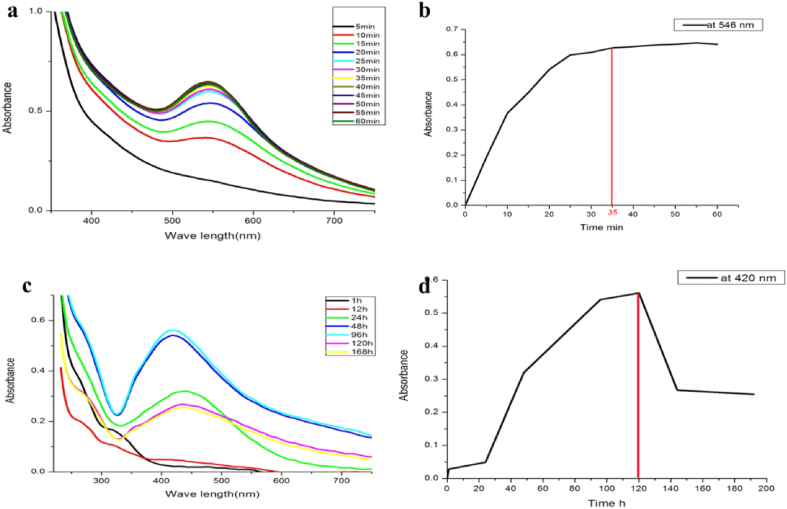
UV–visible spectra for AuNPs (a, b) and AgNPs (c, d) at different contact times and the relation between time and maximum absorbance at 546 nm.

Fig. 3a and b shows the UV–Vis spectroscopy of AuNPs at diverse contact times (5–60 min). Increasing the contact time makes the peak at 546 nm sharper. AuNP formation began within 5 min and increased up to 35 min, but only a small variation can be observed afterward. In contrast, AgNPs synthesized using *Carallia brachiata* leaf extract were saturated in the reduction process after 50 min of the reaction [27]. Fig. 3 c and d show the UV–Vis spectra of AgNPs at various reaction times. Boosting the reaction time gradually increased the absorbance spectrum with SPR at 420 nm, and the color density grew with the incubation duration. This result suggests that the green synthesis of AgNPs is very stable with no aggregation. In past research, the time required to reduce Ag^+^ varied from 24 to 48 h, but the stability was not studied [31]. This finding is slightly similar to those reported previously [32], where AgNPs synthesized using *Tectona grandis* showed a sharp peak after 24 h incubation, indicating mono-dispersity and stability of the AgNPs with no aggregation.

**Fig. 4:**
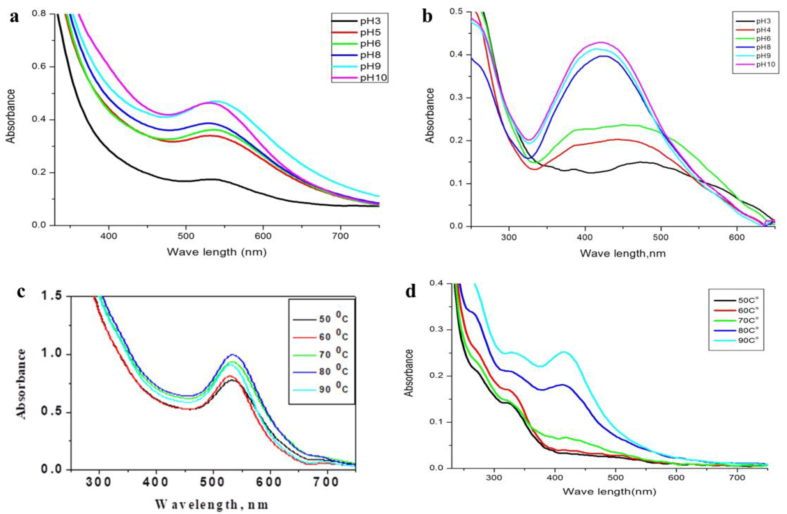
Effect of Teak extracts solution pH and temperature on forming AuNPs (a, c) and AgNPs (b, d).

Fig. 4a and b shows the effect of the pH parameter (3–10) of the Teak extract utilized in the UV–Vis of synthesized NPs. pH parameter is an essential agent affecting the composition, shape, and size of the NPs. It can be seen that the absorbance increases as the pH increases from 3 to 10, with a blue shift in the spectra proposing that the reduction rate of Au^3+^ and Ag ^+^ ions increases with increasing pH (9 and 10). The alkaline environment stimulated a rapid reduction rate of Au^+^, decreased anisotropic growth, and enhanced homogeneous nucleation. On the contrary, a low reduction rate in acidic environments results in heterogeneous nucleation [27]. However, a previous study reported that AgNPs synthesized using *Gmelina arborea* showed an insignificant amount at pH 3, high-yield AgNPs with small sizes at pH 7, and clustered particles at pH 10 [33].

Fig. 4c and d shows UV–visible spectra of the AuNPs and AgNPs prepared at temperatures 50 to 90 °C. The temperature showed effective control in the size and shape of AuNPs and AgNPs. It can be realized that the absorbance peak of AuNPs and AgNPs increased with increasing temperature up to 80 °C allowing the formation of small-sized AgNPs, which disagreed with the previous result that showed a sharp peak at 50, 60, and 80 °C [34,35]. The direct effect of temperature on the size and shape of NPs has been reported by numerous researchers [28,[35], [36], [37]].

In conclusion, the optimized reaction conditions for synthesizing stable AuNPs were set as a 5 ml concentration of Teak extract, incubation time of 35 min, pH of 9, and temperature of 80–90 °C. Similarly, the optimized reaction conditions for synthesizing stable AgNPs were set as a 0.5 ml concentration of Teak extract, incubation time of 48 h, pH of 9, and temperature of 80–90 °C.

### 3.3 TEM analysis

**Fig. 5:**
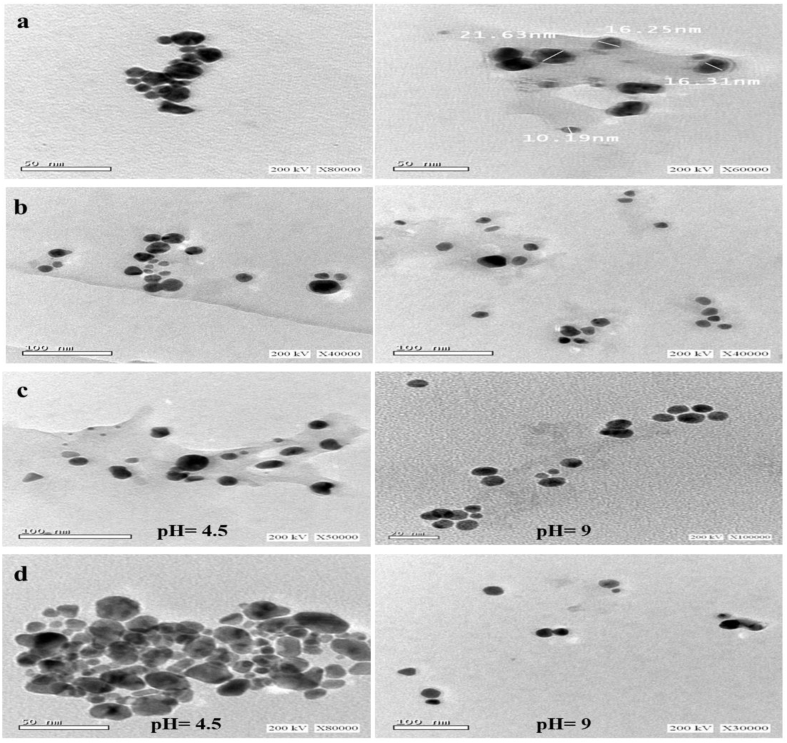
TEM images of (a) AuNPs synthesized using Au^3+^ (2.9 × 10^−4^ M) and 5 ml of Teak extract concentration; (b) AgNPs synthesized using Ag^+^ (1 × 10^−4^ M) and 0.5 ml of Teak extract concentration: (c) AuNPs at pH 4.5 and 9; (d) AgNPs at pH 5 and 9.

TEM figures confirmed the formation and size of the NPs (Fig. 5). For AuNPs, we observed homogeneous size distribution with spherical morphologies and not in agglomerated form. The size of AuNPs was in a range of 10–22 nm (Fig. 5a). TEM shows that AgNPs were spherical with small grains and some dispersed NPs. The AgNP size was between 10 and 30 nm (Fig. 5b). The reduction in the NP size with increasing pH was also detected under TEM for AuNPs and AgNPs at pH 4.5 and 9 (Fig. 5c and d). At pH 10, spherical NPs with sizes less than 20 nm are distinguished (Fig. 5d). The alkaline environment increased the stabilizing and reduction activity of the anti-oxidants in Teak leaf extract [31].

### 3.4 XRD analysis

**Fig. 6:**
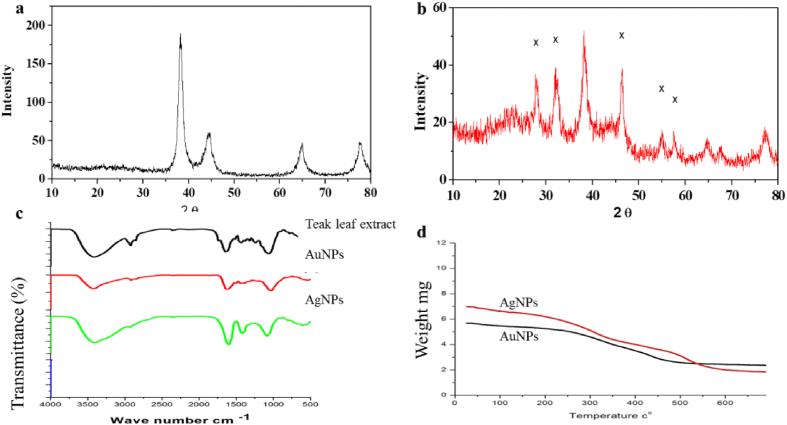
X-Ray diffraction patterns, FTIR spectra, and TGA of capped AuNPs (a, c) and AgNPs (b, d) prepared with aqueous Teak leaf extract.

The synthesized NPs were analyzed utilizing XRD measurements. Fig. 6a shows characteristic peaks at 2θ = 38.29°, 44.43°, and 64.68°, corresponding to (111), (200), and (220) of Au, respectively, indicating that the sample is composed of crystalline gold. The AgNPs pattern was confirmed by detecting 4 unique peaks in XRD (Fig. 6b) at 2θ = 37.85°, 44.0°, 64.2° and 77.2°, corresponding to (111), (200), (220), and (311), respectively, indicating the presence of a face-centered cubic (FCC) structure of metallic silver. Peaks detected in the XRD pattern are similar to those previously reported by Refs. [16,26,30,38]. The XRD results confirmed the crystallization on the surface of the AgNPs by the bio-organic phase.

### 3.5 FTIR analysis

FTIR spectra of Teak extract before and after bio-reduction as shown in Fig. 6c. Several broad bands are observed in the region 500–4000 cm^−1^ centered at 3416.9, 1732, 1608.7, and 1429.2 cm^−1^. Teak leaves have been reported to consist of phenol, flavonoids, quinones, mucilages, and saponins. The broad band at 3416.9 cm^−1^ is caused by the OH groups present in the biomolecules. The band at 2921.1 cm^−1^ refers to stretch carboxylic acids (O–H). The IR bands observed at 1429.2 and 1732.3 cm^−1^ in the teak extract are characteristic of the carbonyl functional group's C–O and C=O stretching modes in ketones, aldehydes, and carboxylic acids. The assignment at 1642.5 cm^−1^ refers to the amide II stretching frequency. The band at 1608.7 cm^−1^ can be attributed to the vibrational modes of aromatic C=C double bonds present in these molecules [39].

For AuNPs, after the bioreduction of Au^3+^ with Teak extract, the shift in the carbonyl bands to 1732.3 cm^−1^ and 1416.7 cm^−1^ refers to the binding of the NPs with the carbonyl group. The hydroxyl band at 3416.9 cm^−1^ shifted to 3423.9 cm^−1^ and 1642.5 cm^−1^ to 1617.9 cm^−1^, indicating Au^3+^ ions were reduced by hydroxyl groups in the flavonoid and then this group oxidized to carbonyl groups [27]. For AgNPs (Fig. 6c), a shift in the absorbance was observed, and the band at 3410.8 cm^−1^ was assigned to the O–H group. The presence of primary amines (N–H bend) can be observed at 1602.4 cm^−1^. The band at 2928.1 cm^−1^ refers to O–H stretch carboxylic acids and 1421.2 cm^−1^, The 1421.2 cm^−^^1^ band indicates that the hydroxyl and carboxylate groups of the extract are bound with silver ions, which suggests that the biological molecules may play a role in forming and stabilizing AgNPs in the aqueous media [30].

### 3.6 TGA analysis

TGA was used to calculate the mass of extract particles present with AgNPs and AuNPs. The temperature decreased significantly in the range of 40–650 nm absorbance, which depended on the type of NPs. For AuNPs, Fig. 6d shows weight loss in the temperature of 50° to 700 °C occurred in 3 places. The first place appeared around 200 °C, the second region around 350 °C, and the third region around 500 °C, referring to 48 % of total weight loss. For AgNPs, weight loss also happened in 4 places, the first at 180 °C, the second at around 320 °C, the third at around 480 °C, and the fourth at around 550 °C, giving 71 % of total weight loss.

### 3.7 DLS analysis

**Fig. 7:**
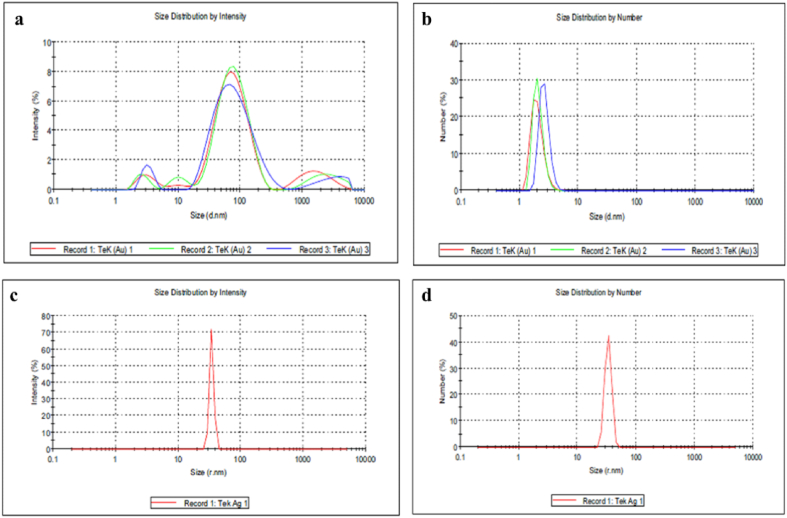
The intensity and number distribution of AuNPs (a and b) and AgNPs (c and d) versus particle diameter in nm.

DLS is most commonly used to analyze NPs and calculate the mean hydrodynamic size and distributions of NPs coated with phytochemicals (Fig. 7). Fig. 7a shows the intensity distribution regarding % intensity as a particle diameter function. The AuNPs provided an overall z-average size of 59 nm with a poly disparity index of 0.421, indicating that the distribution consisted of a monomeric mode without aggregates. The average of three repeated measurements revealed an average peak intensity value of 92 nm. The DLS measurements of AgNPs (Fig. 7c and d) reveal a z-average size of 34 nm, and the number mean for the AgNPs was 100 nm. In the DLS technique, using Mie's theory, it is possible to convert the volume distribution by density into the volume distribution by number or mass (Fig. 7b). Mie theory needs information about the optical features (refractive index and absorption) of the dispersing medium to do transformation.

### 3.8 Cytotoxicity activity of Teak extracts, AuNPs, and AgNPs

The association between cell viability and drug concentrations was designed to calculate IC_50,_ as shown in Table 1. The anticancer activity of aqueous extract (>50 mg/ml) and AuNPs (44.1 mg/ml) are cyto-compatible, while the AgNPs showed the highest anticancer activity with IC_50_ of 6.17 mg/ml, which may be due to modifications in their physic-chemical properties of functional groups of phosphate, proteins, and nitrogen bases groups with silver ions, they may affect various cells through distinct cellular processes [12,40].

**Table 1:** The IC_50_ value of *Tectona grandis* aqueous extract, AuNPs, and AgNPs against HepG2 cell lines.

Treatment	IC_50_ (mg/ml)
Teak	>50
TeakAuNPs	44.1
TeakAgNPs	6.17
Doxorubicin hydrochloride	1.2

Similarly, the *Acalypha indica-*mediated AuNPs showed potential cytotoxicity against HepG2 cell lines at IC_50_ of >50 mg/ml, while AgNPs showed the strongest cytotoxicity at IC_50_ of 11.4 μg/ml [24]. Further, the current study agrees with a previous report that showed the anticancer activity of *Marsdenia tenacissima*-mediated AuNPs against HepG2 at IC_50_ of 59.62 μg/ml. Plant-mediated AuNPs typically stimulate reactive oxygen species (ROS), which cause apoptosis. ROS perturbs signal transduction pathways and induces apoptosis [41]. In contrast, AgNPs biosynthesized using *Momordica cymbalaria* fruit extracts exhibited an IC_50_ of 100 μg/ml against the skeletal muscle L6 Rat cell-line [17]. Previously, several studies reported the anticancer activity of biosynthesized AgNPs with plant extract on A549 cells [28], 3T3 cells [42], and MDA-MB-231 [43].

### 3.9 Antimicrobial activity

**Fig. 8:**
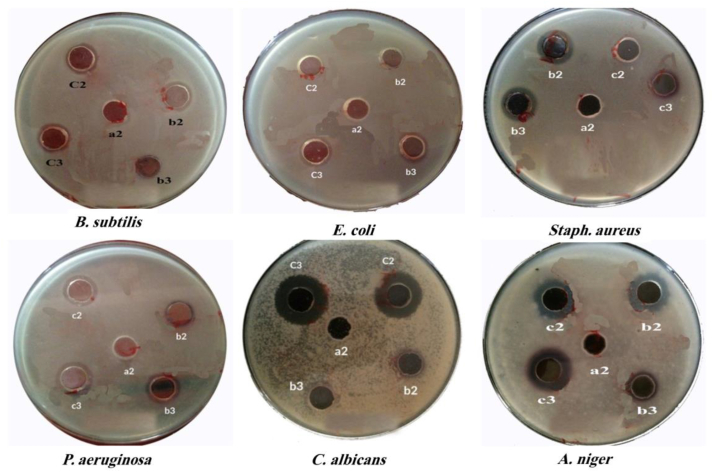
Anti-microbial activities of Teak leaf extract, AuNPs, and AgNPs (at different concentration) against *G* + *bacteria (B. subtilis* and *E. coli), G-bacteria (Staph. aureus* and *P. aeruginosa), and Fungi (C. albicans* and *A. niger).* (a2) = 6 % extract only; b2 = 1.2 % extract+ 1.45 × 10^−3^ M Au+3; b3 = 6 % extract+2.9 × 10^−3^ M Au^+3^; c2 = 1.2 % extract+5 × 10 ^−3^ M Ag^+^; and C3 = 6 % extract+1 × 10 ^−4^ M Ag^+^.

The inhibition zone was measured (mm) to determine the antimicrobial activity of Teak extracts, AuNPs, and AgNPs, as shown in Table 2 and Fig. 8. Teak leave extract at concentrations of 1.2 and 6 % w/v showed no activity against all tested bacteria. Likewise, AuNPs (1.2 % extract and 1.45 × 10^−3^ Au^3+^) showed no antimicrobial activity, possibly due to lipid solubility being the main factor that determines the anti-fungal activity of NPs. The cell membrane regulates the permeability of lipid-soluble material into the cell, and the enzymes of microorganisms block the metal-binding sites [44]. However, increasing Au^3+^ concentration (6 % Teak extract and 2.9 × 10^−3^ M Au^3+^) showed anti-bacterial activity against *P. aeruginosa*, *E. coli*, *B. subtilis*, and *A. niger*. The antibacterial activity of AgNPs at various concentrations showed antibacterial activity against all tested microorganisms. In conclusion, AuNPs (6 % Teak extract and 2.9 × 10^−3^ M Au^3+^) showed significant antibacterial and antifungal activity against *P. aeruginosa* (11 mm)*, A*. *niger* (12 mm), *B*. *subtilis* (12.5 mm), and *E*. *coli* (15.5 mm) as compared to other treatments. This activity may be due to the high yield of the capped AuNPs. Indeed, the conjugation of biomolecules with Au NPs has been found to reduce the growth of bacterial cells [45]. The antimicrobial activity of AuNPs agreed with a previous work reported by Piktel et al. [46], Zhu et al. [47], and Baran et al. [48], but unlike another study by Lomelí-Rosales et al. [49]. AgNPs showed good antifungal activity against *C. albicans* (19 and 20 mm) compared to other treatments. Antifungal activities of AgNPs against *A. niger* have been reported widely by Vivek et al. [50] and Phull et al. [44].

**Table 2:** Anti-microbial activities of Teak leaf extract, AuNPs, and AgNPs (at various concentrations) against G+ bacteria *(B. subtilis* and *E. coli), G-bacteria (Staph. aureus* and *P. aeruginosa), and Fungi (C. albicans* and *A. niger)*.

*Test Organism/*Sample final concentrations	Mean Diameter Of Inhibition Zones (mm)					
	*B. subtilis*	*E. coli*	Staph. aureus	*P. aeruginosa*	C. albicans	*A. niger*
1.2 % extract only	0	0	0	0	0	0
6 % extract only	0	0	0	0	0	0
AuNPs (1.2 % extract and 1.45 × 10^−3^ M Au^3+^)	0	0	0	0	0	0
AuNPs (6 % extract and 2.9 × 10^−3^ M Au^3+^)	12.5	15.5	0	11	0	12
AgNPs (1.2 % extract and 5 × 10^−3^ M Ag^+^)	0	14	16	13.5	19	18.5
AgNPs (6 % extract and 1 × 10^−4^ M Ag^+^)	13	15	14	14.5	20	16.5

## 4 Conclusion

In conclusion, an eco-friendly, safe, cheap process was applied to synthesize AgNPs and AuNPs using the leaf extracts of *Tectona grandis* as a reducing agent. The optimum reaction parameters were measured to prepare stable NPs with small sizes, the best concentration of Teak extracts used to prepare NPs, incubation time, pH, and temperature because several researchers confirmed the effectiveness of the smaller NPs in medical applications. The optimum UV–Vis spectrum for AuNPs and AgNPs was detected at an absorbance of 546 and 420 nm, respectively. TEM and EDS analysis revealed that AuNPs and AgNPs were spherical particles in the range of 10–60 nm and 10–40 nm, respectively. The XRD peaks confirmed the crystalline nature of NPs. The study proved the anticancer effect of AgNPs against HepG2 and the anti-microbial activity of AuNPs against *B. subtilis, E. coli, P. aeruginosa*, and *A. niger*. Teak extracts-mediated NPs are a promising alternative to therapy use in various biological applications and effectively solve the issues resulting from resistance drugs and mutation. The main limitations of this study are that the NPs possess certain physical and chemical characteristics such as composition, size, and shape that greatly influence their properties, including their potential benefits to human health. Thus, optimizing the parameters during the synthesis of AuNPs and AgNPs is crucial. Further, the dose of the NPs could restrict work in-vivo and clinical systems. However, it is important to note that metal NPs can also be toxic, posing a serious risk to both human health and the environment. For instance, prolonged exposure to silver can result in bluish-grey skin and eye discoloration.

## Ethical approval

Not applicable.

## Funding sources

Not applicable.

## Data availability statement

Data will be made available on request.

## CRediT authorship contribution statement

**Huda Mahdi Younis:** Conceptualization, Data curation, Investigation. **Hanaa Ali Hussein:** Conceptualization, Formal analysis, Project administration, Writing – original draft. **Fatin L. Khaphi:** Conceptualization, Writing – original draft, Writing – review & editing. **Zahra Kadhum Saeed:** Conceptualization, Project administration, Writing – original draft.

## Declaration of competing interest

The authors declare that they have no known competing financial interests or personal relationships that could have appeared to influence the work reported in this paper.

## Abbreviations

NPs: nanoparticles
AuNPs: gold NPs
AgNPs: silver NPs
TEM: Transmission-Electron Microscopy
XRD: X-Ray diffraction analysis
FTIR: Fourier transform-infrared spectroscopy
TGA: Thermal-gravimetric-analysis
DLS: Dynamic-light scattering
